# Rose Bengal-Modified Upconverting Nanoparticles: Synthesis, Characterization, and Biological Evaluation

**DOI:** 10.3390/life12091383

**Published:** 2022-09-05

**Authors:** Mykhailo Nahorniak, Ognen Pop-Georgievski, Nadiia Velychkivska, Marcela Filipová, Eliška Rydvalová, Kristýna Gunár, Petr Matouš, Uliana Kostiv, Daniel Horák

**Affiliations:** 1 Institute of Macromolecular Chemistry, Czech Academy of Sciences, Heyrovského nám. 2, 162 06 Prague, Czech Republic; 2 Center of Advanced Preclinical Imaging, First Faculty of Medicine, Charles University, Salmovská 3, 120 00 Prague, Czech Republic

**Keywords:** upconverting, nanoparticles, Rose Bengal, reactive oxygen species, cytotoxicity, photodynamic therapy

## Abstract

High-quality upconverting NaYF_4_:Yb^3+^,Er^3+^ nanoparticles (UCNPs; 26 nm in diameter) based on lanthanides were synthesized by a high-temperature coprecipitation method. The particles were modified by bisphosphonate-terminated poly(ethylene glycol) (PEG) and Rose Bengal (RB) photosensitizer. The particles were thoroughly characterized using transmission electron microscopy, dynamic light scattering, thermogravimetric analysis, FTIR, and X-ray photoelectron and upconversion luminescence spectroscopy in terms of morphology, hydrodynamic size, composition, and energy transfer to the photosensitizer. Moreover, the singlet oxygen generation from RB-containing UCNPs was investigated using 9,10-diphenylanthracene probe under 980 nm excitation. The cytotoxicity of UCNPs before and after conjugation with RB was evaluated on highly sensitive rat mesenchymal stem cells (rMSCs) and significant differences were found. Correspondingly, consi-derable variations in viability were revealed between the irradiated and non-irradiated rat glioma cell line (C6) exposed to RB-conjugated UCNPs. While the viability of rMSCs was not affected by the presence of UCNPs themselves, the cancer C6 cells were killed after the irradiation at 980 nm due to the reactive oxygen species (ROS) production, thus suggesting the potential of RB-conjugated PEG-modified UCNPs for applications in photodynamic therapy of cancer.

## 1. Introduction

Lanthanide-based upconverting nanoparticles (UCNPs) with superb optical properties have attracted a lot of interest in catalysis [1], energy conversion [2], security systems [3], or biomedical applications [4,5]. In biomedicine, they have shown much promise both for diagnostic and therapeutic applications, such as bioimaging and sensing [6], drug delivery [7], or photothermal and photodynamic therapy (PDT) [8,9,10], where the UCNPs perform better than the conventional fluorescent biomarkers [11]. All this is possible due to the unique ability of UCNPs to convert low-energy near-infrared (NIR) irradiation into high-energy ultraviolet and visible light via an anti-Stokes emission mechanism. If the UCNPs are codoped with Yb and Er ions, they emit green and red light, while the particles codoped with Yb and Tm ions emit UV, blue, and NIR light after NIR irradiation. The advantage of NIR light for excitation consists of its ability to penetrate deeper into the living tissues than the visible light [12]. Other beneficial properties of UCNPs include narrow emission peaks, broad Stokes shifts, absence of autofluorescence, low toxicity, and relatively good chemical stability [13].

In the biomedical applications, PDT represents a promising approach to treat various types of cancer. For the cancer treatment, the UCNPs are utilized as NIR acceptors that subsequently emit energy deep in tissue to activate the PDT drug (photosensitizer), which in turn generates reactive oxygen species (ROS). ROS, mostly singlet oxygen (^1^O_2_), then damages the target cancer cells [14,15]. The cytotoxic effects of PDT stem from the oxidation of a large range of biomolecules inside the cells, including nucleic acids, lipids, and proteins, leading to severe alteration of cell signaling cascades or regulation of gene expression [16,17]. As a result, autophagy, apoptosis, or necrosis is induced [18].

The UCNPs can be prepared via three main approaches: coprecipitation [19], thermal decomposition [20], or hydrothermal/solvothermal technique [21]. Other synthetic methods involve polyols [22], *N*-(2-hydroxyethyl)ethylenediamine [23], microwave-assisted synthesis [24], etc. [25]. All the mentioned protocols use organic solvents and thus the obtained UCNPs are hydrophobic; however, they should be hydrophilic and water-dispersible, if used in bioassays or for cell imaging and labeling. To provide sufficient colloidal stability to the UCNPs in water and biologically relevant media, the post-synthesis or in situ surface modification is required, which still remains to be a challenge [26]. Examples of these techniques include chemical modification of hydrophobic surface (e.g., oxidation of oleate or reaction with silanes), replacement of the original ligand by another one, or addition of a thin protective shell on the particles [27]. The latter involves the adsorption of amphiphilic and/or hydrophilic polymers [28], e.g., poly(acrylic acid), polyethyleneimine, poly(ethylene glycol) (PEG), poly(*N*-vinylpyrrolidone), or coating of UCNPs with silica, titanium oxide, gold, or silver [29,30].

As a critical point in the preparation of UCNPs applicable for PDT is to guarantee the efficient energy transfer between the UCNPs and photosensitizer. It can be achieved by several approaches: (i) the selection of a suitable photosensitizer represented by synthetic dyes [31], and/or natural products [32], such as porphyrins [33,34], chlorins [35,36], bacteriochlorins [37], and phthalocyanines [38]; (ii) the suitable combination of UCNP emission with photosensitizer absorption; (iii) the minimal distance between the UCNP and photosensitizer to facilitate efficient absorption of photons produced by UCNPs. The chemical structure of photosensitizer itself may also differ according to PDT application, i.e., cancer versus microbial treatment. The cancer treatment requires a rather lipophilic and electroneutral photosensitizer that can be excited by NIR or far-red light to achieve a good tissue penetration depth [38]. On the other hand, the photosensitizer intended for bacteria killing should have a positive charge and be excitable by visible light [39]. To combine benefits of anticancer and antibacterial photosensitizers in one molecule, Rose Bengal (RB) was suggested [40,41]. RB is a photoactive dye of xanthene type, differing from fluorescein (the most popular synthetic fluorophore) or erythrosine only in substitution of aromatic ring with halogens [42]. RB is easily accessible, with low general toxicity, and possessing antimicrobial properties [43,44]. Its interaction with cells showed promising results for use in PDT [45]. The absorption maximum of RB at 500–570 nm matches well with the most intensive green emission band from UCNPs (520–570 nm) containing Yb^3+^ and Er^3+^ ions, thus making RB a suitable photosensitizer to be used in tissue bonding and anticancer therapy [46,47]. The energy transfer between UCNPs and Rose Bengal and the subsequent ROS generation has been described in [48].

This study presents synthesis and surface engineering of colloidally stable NaYF_4_:Yb^3+^,Er^3+^ UCNPs with immobilized RB photosensitizer with focus on the production of ROS. The ROS generation was detected by 9,10-diphenylanthracene-based spectrophotometric assay. To tightly bind RB to the UCNPs, the dye was modified with phosphonate groups via their reaction with diethyl 2-bromoethylphosphonate. This was followed by the attachment of PEG-alendronate (PEG-Ale) to render colloidal stability to the particles, the general cytotoxicity of which was investigated on rat mesenchymal stem cells (rMSCs). Finally, the cytotoxic effect of RB-conjugated UCNPs on the viability of rat glioma C6 cells after irradiation at 980 nm was demonstrated.

## 2. Materials and Methods

Chemicals: Octadec-1-ene (OD; 90%), sodium hydroxide (99%), ammonium fluoride (99.9%), erbium(III) chloride hexahydrate (98%), anhydrous yttrium(III) and ytterbium(III) chlorides (99%), Rose Bengal disodium salt (RB; 95%), sodium alendronate (Ale; 99%), diethyl 2-bromoethylphosphonate (97%), iodotrimethylsilane (97%), and 9,10-diphenylanthracene (DPA; analytical standard) were purchased from Sigma-Aldrich (St. Louis, MO, USA). Gibco™ phosphate-buffered saline (PBS) and Dulbecco’s modified Eagle’s Medium (DMEM) were obtained from Thermo Fisher Scientific (Waltham, MA, USA). Oleic acid (OA; 98%) and other solvents were purchased from Lachema (Brno, Czech Republic). Phosphate buffer (PB) was prepared from sodium phosphate dibasic dihydrate and sodium phosphate monobasic monohydrate (both Sigma-Aldrich). PEG_5,000_-alendronate (PEG-Ale) was synthesized according to earlier report [49]; its ^1^H NMR spectrum was shown in the Supplementary Materials (Figure S1). Absolute ethanol and other chemicals were obtained from LachNer (Neratovice, Czech Republic). Cellulose dialysis membranes (MWCO 3.5, 14, and 100 kDa) were purchased from Spectrum Europe (Breda, The Netherlands). Water used throughout the study was purified on a Milli-Q IQ 7000 system from Millipore (Molsheim, France).

Cell Lines: Adipose-derived rat mesenchymal stem cells (rMSCs) were kindly gifted by Dr. Pavla Jendelová from the Institute of Experimental Medicine, Academy of Sciences of the Czech Republic, Prague. The C6 glioma cell line was kindly provided by Dr. Čestmír Altaner (Biomedical Research Center SAS, Bratislava, Slovakia). The cells were cultivated in DMEM supplemented with 10% fetal bovine serum (FBS) and 1% penicillin-streptomycin at 37 °C under humidified 5% CO_2_ atmosphere. The cells were passaged after reaching 80–90% confluence using Gibco™ trypsin-EDTA solution.

Preparation of NaYF_4_:Yb^3+^,Er^3+^ Nanoparticles (UCNPs): Lanthanide chlorides (YCl_3_/YbCl_3_/ErCl_3_·6H_2_O = 1.56:0.4:0.04 mol/mol/mol) were charged in a 50-mL three-neck round-bottom flask, OD (15 mL) and OA (6 mL) were added, and the mixture was heated at 160 °C and kept at this temperature for 30 min with magnetic stirring and under argon atmosphere. Afterwards, the mixture was cooled down to room temperature (RT), dispersion of NaOH and NH_4_F (2.5/4 mol/mol) in methanol (10 mL) was dropwise added, and the solution was slowly heated to 120 °C to evaporate methanol and water. The flask with the reaction mixture was then equipped with Vigreux distillation column and the reaction continued at 300 °C for 90 min with stirring. The resulting UCNP@OA particles were se-parated by centrifugation (3460 rcf) for 30 min and washed with hexane/ethanol mixture (1/4 *v*/*v*) four times to remove OA. Before transferring the particles in water, they were washed with water/ethanol solution three times, gradually displacing ethanol by water, until the foam disappeared, and separated by centrifugation (3460 rcf); the resulting particles were dispersed in water and denoted as bare UCNPs.

**Figure 1:**
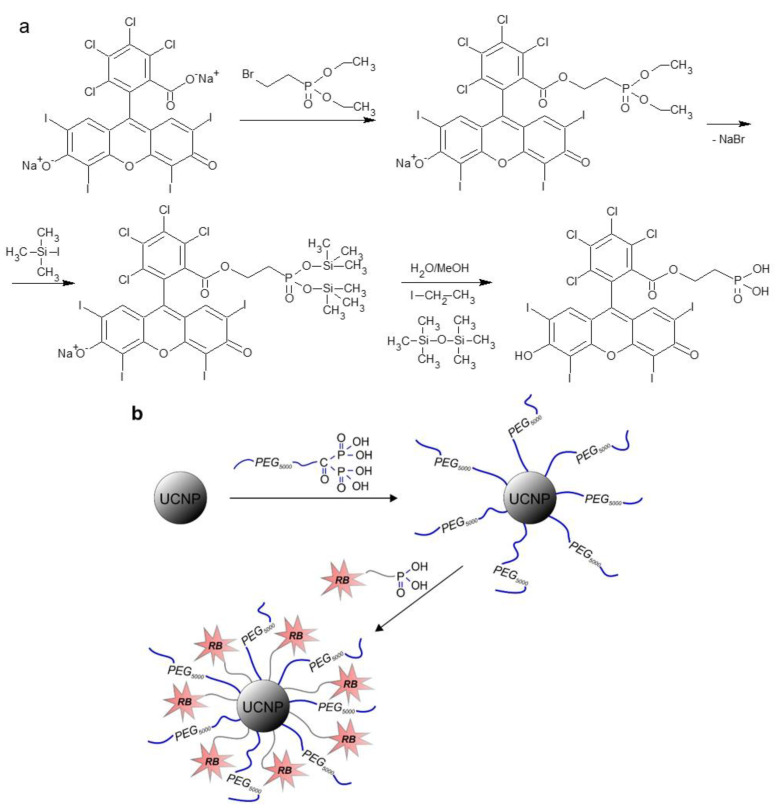
(**a**) Preparation of Rose Bengal-ethylphosphonic acid (RB-PH); (**b**) modification of UCNPs with PEG-Ale and RB-PH.

Preparation of Rose Bengal-Ethylphosphonic Acid (RB-PH): RB was modified with diethyl 2-bromoethylphosphonate, which was followed by the removal of ethoxy groups with iodotrimethylsilane (Figure 1a). In a 10-mL round bottom flask equipped with a reflux condenser, RB (0.1 mmol) and diethyl 2-bromoethylphosphonate (0.3 mmol) were dissolved in dimethylformamide (DMF; 1 mL) at 80 °C for 12 h with magnetic stirring. DMF was then removed at 45 °C under vacuum (1 kPa) and the resulting intermediate was washed with diethyl ether and water three time each and then dried using a rotary evaporator. Next, a solution of iodotrimethylsilane (1 mmol) in methylene chloride/DMF (2/1 *v*/*v*; 1.5 mL) was added dropwise to the above compound and the reaction continued at RT for 20 h with stirring under argon atmosphere. Methanol/water solution (1/1 *v*/*v*; 1 mL) was added and the mixture was kept at RT for 12 h with stirring. The resulting RB-PH was dried at 40 °C on a rotary evaporator under vacuum (8 kPa) and its ^1^H NMR spectrum was shown on Figure S2.

Preparation of UCNP@RB-PH/Ale-PEG: The modification of UCNPs with PEG-Ale and RB-PH was schematically shown on Figure 1b. PEG-Ale (30 mg) was added to aqueous dispersion of bare UCNPs (1 mL; 22.2 mg UCNPs/mL), the mixture was stirred at RT for 12 h, and dialyzed (MWCO 100 kDa) against water for 48 h. The resulting UCNP@Ale-PEG particles (20 mg) were separated by centrifugation (14,100 rcf) for 30 min, resuspended in water (1 mL), RB-PH (5 mg) was added, and the mixture was stirred at RT for 24 h. Excessive RB-PH was removed by dialysis (MWCO 100 kDa) against water/ethanol solution (1:1 *v*/*v*) and water for 48 h each.

Generation of Singlet Oxygen: The generation of ROS, in particular the singlet oxygen, was detected with DPA probe by a Specord 250 Plus UV-Vis spectrophotometer (Analytik Jena; Jena, Germany) according to an earlier described method [50]. Briefly, ethanolic solutions containing 2 × 10^−5^ mol of DPA/l and 1.62 × 10^−9^ mol of RB, RB-PH, or UCNP@RB-PH/Ale-PEG per liter were mixed and irradiated in darkness for 10 min by LED lamp (525–535 nm; 0.16 × 10^−3^ W/mm^2^) and 980 nm laser (MDL-III-980-2W; 0.7 W/cm^2^), respectively. The gradual disappearance of DPA absorbance due to binding of ^1^O_2_ was measured each 10 min at 330–410 nm and the decrease of peaks corresponding to DPA reflected the production of singlet oxygen.

Transmission Electron Microscopy: The size and shape of nanoparticles were investigated by a Tecnai G2 Spirit Twin 12 transmission electron microscope (TEM; FEI; Brno, Czech Republic) [49]. The diameter of nanoparticles and the size distribution were calculated by measuring at least 500 objects from six different TEM micrographs using ImageJ 1.52a software (National Institutes of Health; Bethesda, MD, USA). The number-average diameter (*D*_n_), weight-average diameter (*D*_w_), and dispersity (*Ð*) were calculated using the following equations:

*D*_n_ = ∑N_i_*D*_i_/∑N_i_, (1)

*D*_w_ = ∑N_i_*D*_i_^4^/∑N_i_*D*_i_^3^, (2)

*Ð* = *D*_w_/*D*_n_, (3)

where N_i_ and *D*_i_ are the number and diameter of the measured particle, respectively.

Dynamic Light Scattering (DLS): The hydrodynamic diameter (*D*_h_), size distribution (polydispersity *PD*), and ζ-potential of nanoparticles were measured in water at 25 °C using a ZEN 3600 Zetasizer Nano Instrument (Malvern Instruments; Malvern, UK). *D*_h_ and *PD* were calculated from the intensity-weighted distribution function obtained by CONTIN analysis of the correlation function available in Malvern software v1.32.

Thermogravimetric Analysis (TGA): TGA of particles was performed in air from 25 to 700 °C at a heating rate of 10 °C/min using a PerkinElmer TGA 7 analyzer (Norwalk, CT, USA).

^1^H NMR Spectroscopy: ^1^H NMR spectra of organic compounds in D_2_O or CD_3_OD were recorded at 25 °C with a Bruker Avance III 600 spectrometer (Rheinstetten, Germany) equipped with a 5 mm diffusion probe-head. The conditions included 90° pulse (width 18 μs), relaxation delay 10 s, spectral width 7812 Hz, acquisition time 4.19 s, and 64 scans. During the measurements, temperature was maintained constant within ± 0.2 K using a BVT 3000 temperature unit. The spectra were processed using the Topspin 4.0.5 software from Bruker.

ATR FTIR Spectroscopy: ATR FTIR spectra were recorded on a Thermo Nicolet 870 FTIR spectrometer (Madison, WI, USA) equipped with a liquid nitrogen-cooled mercury cadmium telluride detector using a GoldenGate single refection diamond ATR system (Specac; Orpington, UK).

Luminescence Spectroscopy: Luminescence spectra were recorded using a FS5 spectrofluorometer (Edinburgh Instruments; Edinburgh, UK) coupled with a CW 980 nm laser diode as an excitation source (MDL-III-980-2W) with a maximum laser power 6 W/cm^2^ and a 5 × 8 mm^2^ beam size.

X-ray Photoelectron Spectroscopy (XPS): XPS were measured using a K-Alpha^+^ XPS spectrometer (Thermo Fisher Scientific; East Greenstead, UK) operating at a base pressure of 1 × 10^−7^ Pa. The data were acquired and processed using the Thermo Avantage software. The nanoparticles were spread on a conductive carbon tape and analyzed with microfocused (spot size 400 μm) and monochromated Al Kα X-ray radiation with pass energy of 200 eV for survey and 50 eV for high-energy resolution core level spectra. The X-ray angle of incidence was 30°, the emission angle was along the surface normal, and the dual-charge compensation system employed electrons and low energy Ar^+^ ions. The analyzer transmission function, Scofield sensitivity factors, and effective attenuation lengths for photoelectrons calculated using the standard TPP-2 M formalism were applied for quantification. The binding energy scale of the spectrometer was calibrated by the well-known positions of the C 1s C–C and C–H, C–O and C(=O)–O peaks of poly(ethylene terephthalate) and Cu 2p, Ag 3d, and Au 4f peaks of Cu, Ag, and Au, respectively. All spectra were charge referenced to the C 1s contribution at binding energy of 285 eV attributed to C–C and C–H moieties.

Cytotoxicity Assays: rMSCs were seeded into 96-well flat-bottom plates in concentration 8 × 10^3^ cells/well in 100 μL of complete culture medium. The cells were seeded one day before treatment to spread. The bare UCNPs and UCNP@RB-PH/Ale-PEG (3.5 mg/mL) were diluted in water and the cells were incubated with the particles in concentration range 3.9–500 µg/mL for 24 h at 37 °C. The toxicity of UCNPs was determined by [3-(4,5-dimethylthiazol-2-yl)-2,5-diphenyltetrazolium bromide] (MTT) assay (Abcam; Cambridge, UK), which assesses the cell metabolic activity. Briefly, the medium from growing cells was exchanged by 100 μL of complete growth medium including 10 vol.% of MTT (5 mg/mL) and the plates were incubated at 37 °C for 4 h. After removing the MTT solution, the formazan crystals were dissolved in 100 µL of dimethyl sulfoxide for 10 min. The absorbance was measured at 570 nm on a Synergy Neo plate reader (Bio-Tek; Prague, Czech Republic). The data were expressed as a percentage of viability of bare UCNP-, UCNPs@RB-PH/Ale-PEG-, and RB-treated cells relative to control.

The phototoxic effect of UCNP@RB-PH/Ale-PEG nanoparticles (1 mg/mL) was investigated on glioma C6 cells seeded in concentration 1 × 10^4^ cells/cm^2^ onto 96-well cell culture plate in triplicates. Each well was irradiated at 980 nm for 10 min in a dark room with an MDL-III-980 laser (CMI Laser; Changchun, China) of 2 W power and after 24 h, MTT was performed as previously described with the absorbance measured by a Spark^®^ multimode microplate reader (Tecan; Männedorf, Switzerland). The data were expressed as a percentage of viability of irradiated to non-irradiated cells or irradiated cells incubated with the particles relative to non-irradiated ones.

Statistical Analysis: The cytotoxicity (cell viability) was expressed as the mean ± standard error mean (S.E.M.) of at least three independent experiments performed in triplicates. Two-tailed unpaired Student’s *t*-test was used for the evaluation of statistical differences in both cytotoxicity between bare UCNPs and corresponding RB-conjugated po-lymer-coated nanoparticles and phototoxic effects on cells incubated with and without UCNP@RB-PH/Ale-PEG nanoparticles after laser irradiation. The statistical analysis was calculated using the GraphPad Prism software (version 5.03; San Diego, CA, USA) and the statistically significant values were considered as * *p* < 0.05, ** *p* < 0.01, and *** *p* < 0.001.

## 3. Results and Discussion

### 3.1. Synthesis and Characterization of UCNPs

**Figure 2:**
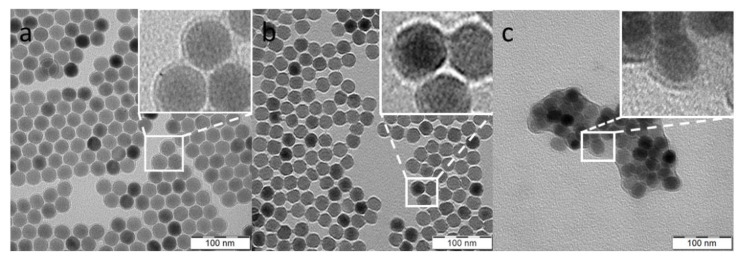
TEM micrograph of (**a**) UCNP@OA; (**b**) UCNP@Ale-PEG; (**c**) UCNP@RB-PH/Ale-PEG nanoparticles.

**Figure 3:**
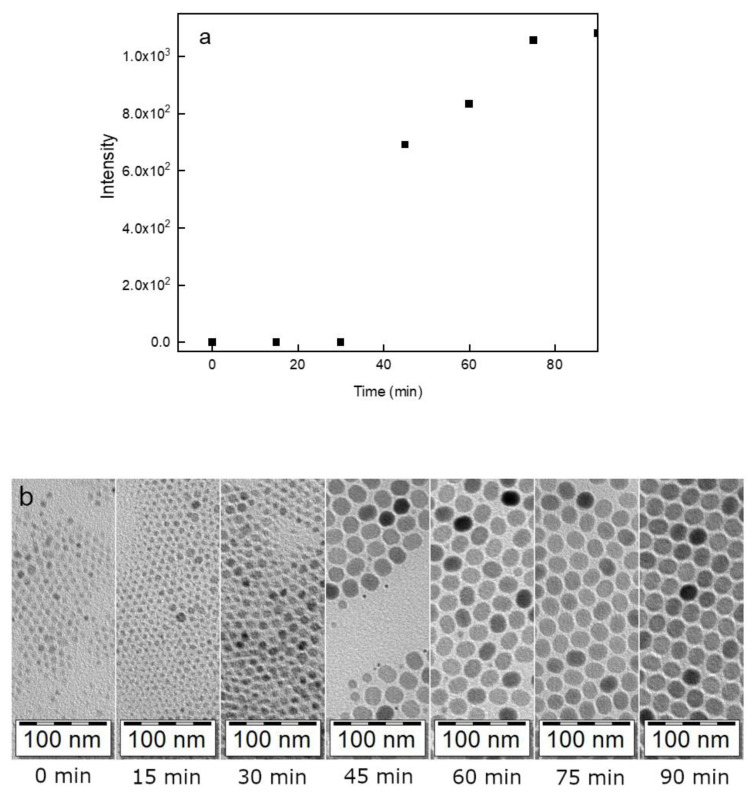
(**a**) Intensity of luminescence of UCNP@OA particles after excitation at 980 nm; (**b**) TEM micrographs of particles prepared at different reaction times (15, 30, 45, 60, 75, and 90 min)/300 °C.

The initial UCNP@OA particles were prepared by a high-temperature coprecipitation of the respective lanthanide chlorides in OD as a solvent and in the presence of OA as a stabilizer [49]. TEM micrographs of the particles showed their spherical shape with number-average diameter *D*_n_ = 26 nm and dispersity *Ð* = 1.01 suggesting uniformity (Figure 2a; Table 1). The corona (~1 nm) was clearly visible on the micrograph, confirming the presence of OA on the particle surface. The TEM analysis was combined with the upconversion luminescence spectroscopy under NIR excitation at 980 nm to observe the formation of UCNPs at different reaction times (Figure 3). At a short reaction time (up to 20 min), the intensity of luminescence was negligible (Figure 3a) and it was accompanied with the formation of nuclei (Figure 3b). However, after 25-min reaction, the intensity of emitted light increased rapidly with the nuclei growing due to Ostwald ripening [51]. As the coprecipitation proceeded, the nuclei growth was completed with the formation of mature particles. This process did not finish until the reaction time reached 80 min; afterwards, the particle size and shape did not change. Based on this observation, the reaction time of 90 min was selected in further experiments to ensure complete formation of UCNP@OA particles, their stable morphology, and reproducible results.

**Figure 4:**
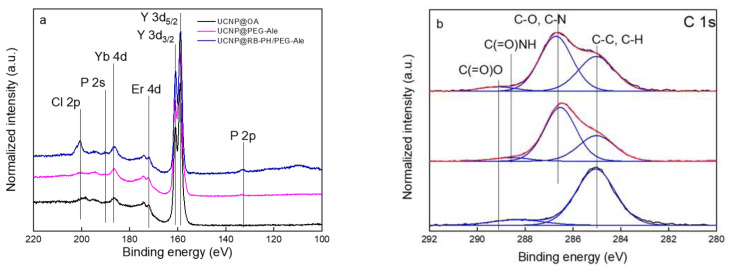
Comparison of high-resolution XPS spectra of initial UCNP@OA (black), UCNP@Ale-PEG (magenta), and UCNP@RB-PH/Ale-PEG (royal) in the region of (**a**) P 2p, Y 3d, Er 4d, Yb 4d, P 2s, Cl 2p and (**b**) C 1s (red, fitted data; blue, individual contributions of functional groups on the UNCP surface).

The composition of UCNP@OA particles was monitored in XPS spectra, showing the dominant double Y 3d peak at 159.5 eV, minor contributions of Er 4d and Yb 4d at 172.5 eV and 186.9 eV, respectively, and F 1s and Na 1s peaks at 686.8 and 1071.1 eV, respectively (Figure 4a; Table 2); the results agreed with the earlier published data [52]. In the C 1s spectrum, two peaks originating from C–C and C(=O)–O^−^ groups documented the presence of OA stabilizer on the particle surface (Figure 4b). High-resolution XPS spectroscopy thus confirmed the chemical structure of UCNPs. FTIR spectrum of UCNP@OA particles also showed characteristic OA peaks (Figure S3). Specifically, C-H asymmetric and symmetric stretching vibrations (2924 and 2853 cm^−1^), C=O stretching vibration (1712 cm^−1^), and CH_2_ bending vibration (1462 cm^−1^). Thus, the XPS and FTIR results confirmed the presence of OA on the UCNP@OA particles.

### 3.2. Surface Engineering of UCNPs

As the UCNP@OA particles were hydrophobic due to the presence of oleic acid on the surface, as well as lacking any immobilized sensitizer, their surface had to be modified to render them with colloidally stability in aqueous biological media without aggregation during storage; at the same time, sufficient amounts of sensitizer should be attached. One way was to anchor the carboxyl-RB photosensitizer on poly(allylamine)-modified UCNPs via carbodiimide chemistry [53].

**Figure 5:**
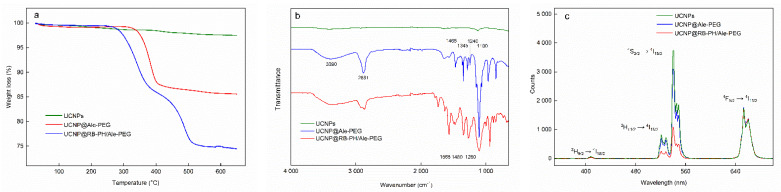
(**a**) Thermogravimetric analysis; (**b**) FTIR; (**c**) luminescence spectra of surface-modified UCNPs excited at 980 nm.

Here, the UCNP@OA particles were washed with hexane/ethanol and water/ethanol to remove OA and transferred in water to obtain bare UCNPs, which were subsequently modified with PEG-Ale and RB-PH according to the protocol described in Materials and Methods. PEG-Ale was bound to the UCNPs by bisphosphonate groups. As the molar mass of RB-PH was lower than that of PEG-Ale, RB-PH molecule could penetrate the spaces between adjacent PEG chains, being attached to the particle surface via phosphonate groups. The modifications were not distinctly contrasted in TEM micrographs of both UCNP@Ale-PEG (Figure 2b) and UCNP@RB-PH/Ale-PEG nanoparticles (Figure 2c); in the latter particles, clusters were formed during drying. Corresponding DLS measurements in water revealed the change of hydrodynamic diameter of particles from 119 nm for bare UCNP to 89 and 215 nm for UCNP@Ale-PEG and UCNP@RB-PH/Ale-PEG, respectively. The relatively low value of *D*_h_ of UCNP@Ale-PEG particles can be explained by their reduced aggregation compared to that of bare UCNPs. On the other hand, the increased *D*_h_ of UCNP@RB-PH/Ale-PEG is related to the presence of thick RB-PH/Ale-PEG shell on the particle surface. At the same time, the ζ-potential of UCNP@Ale-PEG and UCNP@RB-PH/Ale-PEG particles decreased from 36 mV for bare UCNPs to 18 and −10 mV, respectively (Table 1), due to the electroneutrality of PEG and also due to the negatively charged RB [54]. TGA of UCNP@RB-PH/Ale-PEG confirmed the presence of PEG-Ale and RB-PH, amounting to 11.2 and 11.9 wt.%, respectively (Figure 5a). The ATR FTIR spectra of bare UCNPs showed very low characteristic C-H stretching vibrations (2924 and 2853 cm^−1^) of OA, confirming efficient washing of starting UCNP@OA. The FTIR spectrum of UCNP@Ale-PEG particles confirmed the modification with Ale-PEG. The stretching vibration at 3000–3660 cm^−1^ corresponded to the O-H bond, the peaks at 2881, 1465, and 1345 cm^−1^ were attributed to the stretching of C-H, and the bands at 1240 and 1000–1200 cm^−1^ were ascribed to the vibration of C-O-C bond [55,56]. In the spectrum of UCNP@RB-PH/Ale-PEG, the modification of UCNPs with RB-PH was documented by stretching vibrations at ~1565, 1490, and 1460 cm^−1^ ascribed to the characteristic absorption of aromatic ring; the peaks at 1260 cm^−1^ were attributed to C–O–C groups (Figure 5b).

The upconversion luminescence of UCNPs induced by energy transfer, as well as the light absorbance by RB, were investigated by comparison of emission spectra. In brief, the Yb^3+^ ions absorbed photons at 980 nm, inducing transfer of their f-electrons from the ground ^2^F_7/2_ state to the ^2^F_5/2_ excited state (Figure 5c). Consequently, the proximity of energy levels of Er^3+^ and Yb^3+^ ions allowed the energy transfer from excited Yb^3+^ sensitizer to Er^3+^ emitter. Here, two processes could occur: (i) excitation state absorption, where Er^3+^ energy levels (^2^H_11/2_ and ^4^S_3/2_) were pumped up with energy, and (ii) cross-relaxation of Er^3+^ ions pumped up to ^4^F_9/2_ energy level [57]. In the fluorescence spectra, the green emission was characterized by peaks at 515–534 and 534–560 nm corresponding to the ^2^H_11/2_ → ^4^I_15/2_ and ^4^S_3/2_ → ^4^I_15/2_ electron transitions, respectively. The red emission at 640–674 nm was ascribed to the ^4^F_9/2_ → ^4^I_15/2_ transition, while the relatively weak blue emission appeared at 402–412 nm due to ^2^H_9/2_ → ^4^I_15/2_ transitions. The intensities of peaks of bare UCNPs and UCNP@Ale-PEG at a concentration of 1 mg/mL in the excitation region 515–560 nm were almost the same, while that of UCNP@RB-PH/Ale-PEG significantly decreased. This was associated with the light absorption by RB-PH (Figure S4) and the efficient energy transfer from particles to photosensitizer. The excitation curves of bare UCNPs and UCNP@RB-PH/Ale-PEG particles were normalized relative to the area of red peak (630–690 nm), do-cumenting that 72% of excited light was absorbed by RB-PH (Figure 5c).

In the high-resolution P 2p, Y 3d, Er 4d, Yb 4d, P 2s, and Cl 2p XPS spectra of UCNP@RB-PH/Ale-PEG particles, the modification of UCNPs with Ale-PEG and RB-PH increased the phosphorous content; moreover, new bands of iodine and chlorine appeared (Figure 4a; Table 2). At the same time, the presence of RB-PH increased the C-C peak at 285.0 eV in the high-resolution C 1s XPS spectrum of UCNP@RB-PH/Ale-PEG (Figure 4b). The concomitant XPS and DLS analysis thus confirmed the successful formation of RB-PH/Ale-PEG coating around the UCNPs. Considering the size of PEG (5 kDa), RB-PH photosensitizer is supposed to be at the interphase between UCNP and polymer shell. This surface engineering of UCNPs with RB-PH/Ale-PEG shield the particles from unspecific interactions with proteins of body fluids and might diminish fast clearance in in vivo applications, while preserving high activity of RB.

**Figure 6:**
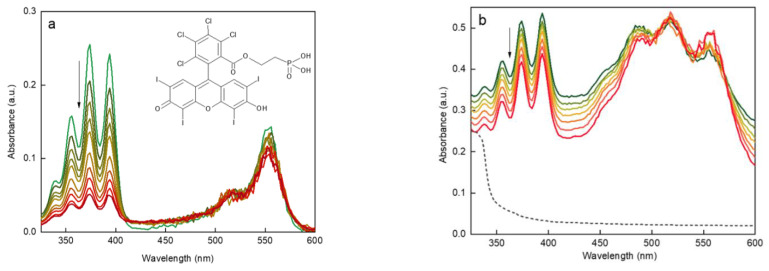
UV-Vis spectra of (**a**) RB-PH excited at 525–535 nm and (**b**) UCNP@RB-PH/Ale-PEG excited at 980 nm document time-dependent decrease of DPA absorbance due to ^1^O_2_ generation (see the arrow); (**b**) black dashed line is for UCNP@OA particles. Each curve was measured after 10 min delay.

RB is known to effectively produce singlet oxygen, when irradiated with green light [58] (Figure S5). ROS production by RB-PH was confirmed by binding ^1^O_2_ to DPA fluorochrome after the excitation at 525–535 nm. The amount of ^1^O_2_ released from RB-PH continuously increased with increasing irradiation time, which correlated with the disappearance of DPA absorbance (Figure 6a). Analogous experiment was performed in complete darkness with UCNP@RB-PH/Ale-PEG particles excited at 980 nm (Figure 6b). The decreasing absorbance of DPA (330–410 nm) confirmed the production of ROS. A noticeably quicker generation of ^1^O_2_ by RB-PH compared to that by UCNP@RB-PH/Ale-PEG was related to the area of irradiation; while the RB-PH was illuminated over the entire surface of cuvette by LED lamp, the UCNP@RB-PH/Ale-PEG particles were illuminated only in the path of the laser beam.

### 3.3. Cytotoxicity of UCNPs

**Figure 7:**
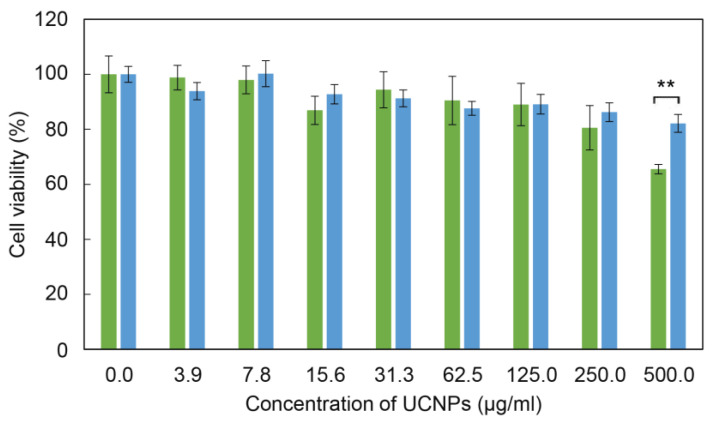
Cytotoxicity of bare UCNPs (green) and UCNP@RB-PH/Ale-PEG particles (blue) against rMSCs after 24 h of incubation using MTT cell viability assay. Error bars represent standard error means (S.E.M.) calculated from at least three different experiments performed in triplicates; ** *p* < 0.01 (two-tailed unpaired Student’s *t*-test).

For the evaluation of general cytotoxicity (without irradiation and the subsequent generation of ROS) of surface-engineered UCNPs, the adult rat stem cells (rMSCs) were selected, which are healthy (non-cancer) multipotent cells, capable of differentiating into multiple cell types; such cells are more sensitive to nanoparticles or drugs than the terminally differentiated cells [59,60]. Higher susceptibility to toxic compounds makes these cells a valuable model for the evaluation of biocompatibility of nanoparticles in their potential applications in vivo. The cytotoxicity was assessed by MTT cell viability assay after 24 h of incubation with rMSCs (Figure 7). The bare UCNPs were nontoxic at all particle concentrations, except for the highest one (500 µg/mL). However, the sensitizer-containing RB-PH/Ale-PEG coating significantly decreased the cytotoxicity of particles at 500 µg/mL that was manifested by the change of the viability from 66 ± 2% to 82 ± 3% for bare UCNPs and UCNP@RB-PH/Ale-PEG (*p* < 0.01), respectively; thus, this concentration can be considered as nontoxic for rMSCs. It is known that the coating of nanoparticles might influence their cytotoxicity, depending on type of coating, cells, etc. [61,62]. Our coating with RB-PH/Ale-PEG favorably decreased cytotoxicity of bare UCNPs, which supports their usefulness for potential biomedical application.

**Figure 8:**
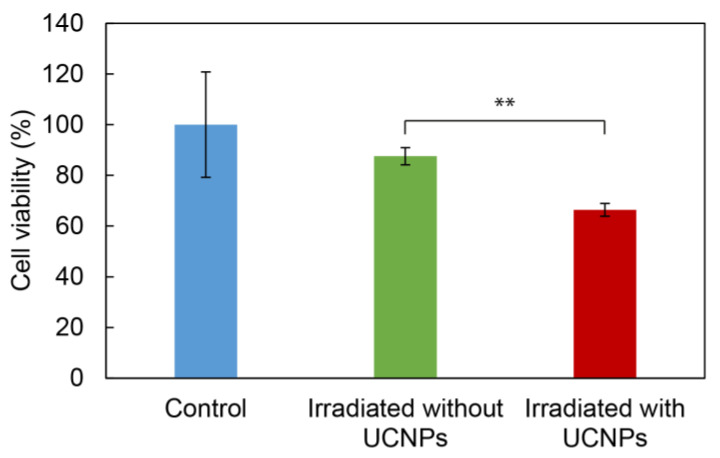
Viability of C6 glioma cells in the presence of UCNP@RB-PH/Ale-PEG particles (1 mg/mL) without irradiation (control; blue) and after 10 min of irradiation at 980 nm in the absence (green) and presence of particles (red); ** *p* < 0.01 (Student’s *t*-test).

The impact of ROS production by UCNP@RB-PH/Ale-PEG nanoparticles manifested as the phototoxic effect was investigated on the C6 glioma cell line after 10 min of irradiation at 980 nm vs. in the absence of irradiation or without UCNP@RB-PH/Ale-PEG nanoparticles. It has already been shown before that the exposure of cells to 980 nm laser irradiation for at least 30 min did not decrease cell viability [63]. The C6 cells were selected as a model to demonstrate the potential therapeutic effect of particles on cancer cells, which should be killed after NIR irradiation. The viability of C6 cells incubated with the UCNP@RB-PH/Ale-PEG particles decreased after the laser irradiation from 88 ± 6% (in the absence of particles) to 66 ± 4% probably due to ROS production after the Rose Bengal activation (Figure 8). Ultimately, the cell experiments showed that even mild general toxicity associated with the highest concentration of bare UCNPs can be advantageously surpassed by the polymeric coating containing RB sensitizer, which conferred the nonto-xicity to UCNPs at the one side and enabled their utilization for the ROS production on the other side. Effective ROS production by the UCNP@RB-PH/Ale-PEG particles (Figure 8) can justify a significant decrease in the cell viability of cancer cells after irradiation.

## 4. Conclusions

This report describes the synthesis of NaYF_4_:Yb^3+^,Er^3+^ upconverting nanoparticles and their modification with RB as a photosensitizer to enable the generation of ROS, preferably, deep in the tissues to kill cancer cells. The synthesis was based on a high-tempe-rature coprecipitation of lanthanide precursors that allowed us to obtain UCNPs via a controllable and reproducible approach. To render the UCNPs with colloidal stability and to decrease the toxicity, the particles were covered with PEG-Ale as a stabilizer that is a well-known FDA-approved hydrophilic agent, suppressing non-specific protein adsorption. Moreover, the newly synthesized RB-PH photosensitizer benefited from the presence of phosphonate groups able to very strongly coordinate with rare earth atoms, thus ensuring strong attachment to UCNPs [64]. The bare UCNPs, especially at the highest concentration (500 µg/mL), were mildly cytotoxic for rMSCs (viability ~70%), while the UCNP@RB-PH/Ale-PEG particles were almost nontoxic (viability ~80%). As the latter particles did not exert toxic effects on highly sensitive rMSCs, we thus demonstrated biocompatibility for a wider range of applications with different cell types, namely, C6 glioma cells that substantially differ from widely applied glioma cell line U87MG [53]. The ROS production by UCNP@RB-PH/Ale-PEG particles after 10 min of irradiation with NIR laser successfully led to the killing of the cancer cells manifested as a decrease in cell viability >20%. Thus, the combination of RB with PEGylated UCNPs represents a promising approach for the treatment of cancer via photodynamic therapy.

## Figures and Tables

**Table 1:** Particle characterization.

Type of Nanoparticles	*D*_n_ ^1^ (nm)	*Ð* ^2^	*D*_h_ ^3^ (nm)	*PD* ^4^	ξ-Potential (mV)
Bare UCNPs	26	1.01	119	0.15	36
UCNP@Ale-PEG			89	0.18	18
UCNP@RB-PH/Ale-PEG			215	0.35	−10

^1,2^ Number-average diameter and dispersity by TEM; ^3,4^ hydrodynamic diameter and polydispersity by DLS, respectively.

**Table 2:** Surface composition of UCNP@OA, UCNP@Ale-PEG, and UCNP@RB-PH/Ale-PEG particles (in wt.%) according to XPS spectroscopy.

Element	UCNP@OA	UCNP@Ale-PEG	UCNP@RB-PH/Ale-PEG
P 2p	- *	0.5	0.9
Y 3d	38.7	38.1	34.2
Er 4d	3.2	2.1	1.6
Yb 4d	0.5	0.5	0.3
Cl 2p	-	-	4.1
C 1s C–C, C–H	16.9	5.3	7.7
C 1s C–O, C–N	-	9.3	10.9
C 1s C(=O)–NH	-	-	
C 1s C(=O)–O	2.3	0.7	1.0
N 1s	-	-	-
O 1s	2.9	10.0	9.8
I 3d	-	-	4.4
F 1s	27.3	29.3	22.2
Na 1s	8.2	4.2	2.9

* Below the detection limit of XPS measurement.

## Data Availability

Data can be found with M.N.

## Supplementary Materials

The following supporting information can be downloaded at: https://www.mdpi.com/article/10.3390/life12091383/s1, Figure S1: High-resolution ^1^H NMR spectrum; Figure S2: High-resolution ^1^H NMR spectrum; Figure S3: FTIR spectrum; Figure S4: UV-Vis absorption spectrum; Figure S5: UV-Vis spectra.

## References

[1] Freitag M., Möller N., Rühling A., Strassert C.A., Ravoo B.J., Glorius F. (2019). Photocatalysis in the dark: Near-infrared light driven photoredox catalysis by an upconversion nanoparticle/photocatalyst system. ChemPhotoChem.

[2] Bünzli J.G., Eliseeva S.V. (2010). Lanthanide NIR luminescence for telecommunications, bioanalyses and solar energy conversion. J. Rare Earths.

[3] You M., Zhong J., Hong Y., Duan Z., Lin M., Xu F. (2015). Inkjet printing of upconversion nanoparticles for anti-counterfeit applications. Nanoscale.

[4] Ansari A.A., Parchur A.K., Thorat N.D., Chend G. (2021). New advances in pre-clinical diagnostic imaging perspectives of functionalized upconversion nanoparticle-based nanomedicine, *Coord*. Chem. Rev..

[5] Wang C., Cheng L., Liu Z. (2011). Drug delivery with upconversion nanoparticles for multi-functional targeted cancer cell imaging and therapy. Biomaterials.

[6] Liang G., Wang H., Shi H., Wang H., Zhu M., Jing A., Li J., Li G. (2020). Recent progress in the development of upconversion nanomaterials in bioimaging and disease treatment. J. Nanobiotechnol..

[7] Cheng Z., Lin J. (2015). Synthesis and application of nanohybrids based on upconverting nanoparticles and polymers. Macromol. Rapid Commun..

[8] Luo H., Kong L., Zhang F., Huang C., Chen J., Zhang H., Yu H., Zheng S., Xu H., Zhang Y. (2021). Light-controlled nanosystem with size-flexibility improves targeted retention for tumor suppression. Adv. Funct. Mater..

[9] Hamblin M. (2018). Upconversion in photodynamic therapy: Plumbing the depths. Dalton Trans..

[10] Idris N., Gnanasammandhan M., Zhang J., Ho P., Mahendran R., Zhang Y. (2012). In vivo photodynamic therapy using upconversion nanoparticles as remote-controlled nanotransducers. Nat. Med..

[11] Wang X., Li Y. (2007). Monodisperse nanocrystals: General synthesis, assembly, and their applications. Chem. Commun..

[12] Hudson D.E., Hudson D.O., Wininger J., Richardson B.D. (2013). Penetration of laser light at 808 and 980 nm in bovine tissue samples. Photomed. Laser Surg..

[13] Gorris H., Wolfbeis O. (2013). Photon-upconverting nanoparticles for optical encoding and multiplexing of cells, biomolecules, and microspheres. Angew. Chem. Int. Ed..

[14] Chen G., Qiu H., Prasad P., Chen X. (2014). Upconversion nanoparticles: Design, nanochemistry, and applications in theranostics. Chem. Rev..

[15] Xu J., Shi R., Chen G., Dong S., Yang P., Zhang Z., Niu N., Gai S., He F., Fu Y. (2020). All-in-one theranostic nanomedicine with ultrabright second near-infrared emission for tumor-modulated bioimaging and chemodynamic/photodynamic therapy. ACS Nano.

[16] Mroz P., Yaroslavsky A., Kharkwal G., Hamblin M. (2011). Cell death pathways in photodynamic therapy of cancer. Cancers.

[17] Klotz L., Kröncke K., Sies H. (2003). Singlet oxygen-induced signaling effects in mammalian cells. Photochem. Photobiol. Sci..

[18] Buytaert E., Dewaele M., Agostinis P. (2007). Molecular effectors of multiple cell death pathways initiated by photodynamic therapy. Biochim. Biophys. Acta.

[19] Yi G., Lu H., Zhao S., Ge Y., Yang W., Chen D., Guo L.-H. (2004). Synthesis, characterization, and biological application of size-controlled nanocrystalline NaYF_4_:Yb,Er infrared-to-visible up-conversion phosphors. Nano Lett..

[20] Zhang Y., Sun X., Si R., You L., Yan C. (2005). Single-crystalline and monodisperse LaF_3_ triangular nanoplates from a single-source precursor. J. Am. Chem. Soc..

[21] Zhuang J., Liang L., Sung H., Yang X., Wu M., Williams I.D., Feng S., Su Q. (2007). Controlled hydrothermal growth and up-conversion emission of NaLnF_4_ (Ln = Y, Dy-Yb). Inorg. Chem..

[22] Li C., Zhang C., Hou Z., Wang L., Quan Z., Lian H., Lin J. (2009). β-NaYF_4_ and β-NaYF_4_:Eu^3+^ microstructures: Morphology control and tunable luminescence properties. J. Phys. Chem. C.

[23] Heer S., Kömpe K., Güdel H., Haase M. (2004). Highly efficient multicolour upconversion emission in transparent colloids of lanthanide-doped NaYF_4_ nanocrystals. Adv. Mater..

[24] Panov N., Marin R., Hemmer E. (2018). Microwave-assisted solvothermal synthesis of upconverting and downshifting rare-earth-doped LiYF_4_ microparticles. Inorg. Chem..

[25] Wang M., Abbineni G., Clevenger A., Mao C., Xu S. (2011). Upconversion nanoparticles: Synthesis, surface modification and biological applications. Nanomed. NBM.

[26] Himmelstoß S., Hirsch T. (2019). Long-term colloidal and chemical stability in aqueous media of NaYF_4_-type upconversion nanoparticles modified by ligand-exchange. Part. Part. Syst. Charact..

[27] Muhr V., Wilhelm S., Hirsch T., Wolfbeis O. (2014). Upconversion nanoparticles: From hydrophobic to hydrophilic surfaces. Acc. Chem. Res..

[28] Freij-Larsson C., Nylander T., Jannasch P., Wesslén B. (1996). Adsorption behaviour of amphiphilic polymers at hydrophobic surfaces: Effects on protein adsorption. Biomaterials.

[29] Kamiya H., Iijima M. (2010). Surface modification and characterization for dispersion stability of inorganic nanometer-scaled particles in liquid media. Sci. Technol. Adv. Mater..

[30] Ziental D., Czarczynska-Goslinska B., Mlynarczyk D., Glowacka-Sobotta A., Stanisz B., Goslinski T., Sobotta L. (2020). Titanium dioxide nanoparticles: Prospects and applications in medicine. Nanomaterials.

[31] Wainwright M., Crossley K. (2002). Methylene blue—A therapeutic dye for all seasons?. J. Chemother..

[32] Allison R., Downie G., Cuenca R., Hu X., Childs C., Sibata C. (2004). Photosensitizers in clinical PDT. Photodiagnosis Photodyn. Ther..

[33] Maisch T., Bosl C., Szeimies R., Lehn N., Abels C. (2005). Photodynamic effects of novel XF porphyrin derivatives on prokaryotic and eukaryotic cells. Antimicrob. Agents Chemother..

[34] Zheng B., Zhong D., Xie T., Zhou J., Li W., Ilyas A., Lu Y., Zhou M., Deng R. (2021). Near-infrared photosensitization via direct triplet energy transfer from lanthanide nanoparticles. Chem.

[35] Wagner A., Denzer U., Neureiter D., Kiesslich T., Puespoeck A., Rauws E.A.J., Emmanuel K., Degenhardt N., Frick U., Beuers U. (2015). Temoporfin improves efficacy of photodynamic therapy in advanced biliary tract carcinoma: A multicenter prospective phase II study. Hepatology.

[36] Wang C., Tao H., Cheng L., Liu Z. (2011). Near-infrared light induced in vivo photodynamic therapy of cancer based on upconversion nanoparticles. Biomaterials.

[37] Saavedra R., Rocha L., Dąbrowski J., Arnaut L. (2013). Modulation of biodistribution, pharmacokinetics, and photosensitivity with the delivery vehicle of a bacteriochlorin photosensitizer for photodynamic therapy. ChemMedChem.

[38] Abrahamse H., Hamblin M. (2016). New photosensitizers for photodynamic therapy. Biochem. J..

[39] Sperandio F., Huang Y., Hamblin M. (2013). Antimicrobial photodynamic therapy to kill Gram-negative bacteria. Recent Pat. Anti-Infect. Drug Discov..

[40] Qin J., Kunda N., Qiao G., Calata J.F., Pardiwala K., Prabhakar B.S., Maker A.V. (2017). Colon cancer cell treatment with Rose Bengal generates a protective immune response via immunogenic cell death. Cell Death Dis..

[41] Nakonechny F., Barel M., David A., Koretz S., Litvak B., Ragozin E., Etinger A., Livne O., Pinhasi Y., Gellerman G. (2019). Dark antibacterial activity of Rose Bengal. Int. J. Mol. Sci..

[42] Linden S., Neckers D. (1988). Fundamental properties of Rose Bengal. 25. Bleaching studies of Rose Bengal onium salts. J. Am. Chem. Soc..

[43] Pérez-Laguna V., García-Luque I., Ballesta S., Pérez-Artiaga L., Lampaya-Pérez V., Samper S., Soria-Lozano P., Rezusta A., Gilaberte Y. (2018). Antimicrobial photodynamic activity of Rose Bengal, alone or in combination with Gentamicin, against planktonic and biofilm *Staphylococcus aureus*. Photodiagnosis Photodyn. Ther..

[44] Costa A., Rasteiro V., Pereira C., Rossoni R., Junqueira J., Jorge A. (2011). The effects of Rose Bengal- and erythrosine-mediated photodynamic therapy on *Candida albicans*. Mycoses.

[45] Panzarini E., Inguscio V., Dini L. (2011). Overview of cell death mechanisms induced by Rose Bengal acetate-photodynamic therapy. Int. J. Photoenergy.

[46] Xu N., Yao M., Farinelli W., Hajjarian Z., Wang Y., Redmond R.W., Kochevar I.E. (2014). Light-activated sealing of skin wounds. Lasers Surg. Med..

[47] Panzarini E., Inguscio V., Fimia G., Dini L. (2014). Rose Bengal acetate photodynamic therapy (RBAc-PDT) induces exposure and release of damage-associated molecular patterns (DAMPs) in human HeLa cells. PLoS ONE.

[48] Wang Y., Liu K., Liu X., Dohnalová K., Gregorkiewicz T., Kong X., Aalders M.C.G., Buma W.J., Zhang H. (2011). Critical shell thickness of core/shell upconversion luminescence nanoplatform for FRET application. J. Phys. Chem. Lett..

[49] Kostiv U., Lobaz V., Kučka J., Švec P., Sedláček O., Hrubý M., Janoušková O., Francová P., Kolářová V., Šefc L. (2017). A simple neridronate-based surface coating strategy for upconversion nanoparticles: Highly colloidally stable ^125^I-radiolabeled NaYF_4_:Yb^3+^/Er^3+^@PEG nanoparticles for multimodal in vivo tissue imaging. Nanoscale.

[50] Yang Q., Zhao C., Zhao J., Ye Y. (2017). Synthesis and singlet oxygen activities of near infrared photosensitizers by conjugation with upconversion nanoparticles. Opt. Mater. Express.

[51] Kabalnov A. (2001). Ostwald ripening and related phenomena. J. Dispers. Sci. Technol..

[52] Kostiv U., Farka Z., Mickert M., Gorris H.H., Velychkivska N., Pop-Georgievski O., Pastucha M., Odstrčilíková E., Skládal P., Horák D. (2020). Versatile bioconjugation strategies of PEG-modified upconversion nanoparticles for bioanalytical applications. Biomacromolecules.

[53] Ren J., Ding Y., Zhu H., Li Z., Dai R., Zhao H., Hong X., Zhang H. (2022). Emitter-active shell in NaYF_4_:Yb,Er/NaYF_4_:Er upconversion nanoparticles for enhanced energy transfer in photodynamic therapy. ACS Appl. Nano Mater..

[54] Argüeso P., Tisdale A., Spurr-Michaud S., Sumiyoshi M., Gipson I. (2006). Mucin characteristics of human corneal-limbal epithelial cells that exclude the Rose Bengal anionic dye. Investig. Opthalmol. Vis. Sci..

[55] Samsudin A., Lai H., Isa M. (2014). Biopolymer materials based carboxymethyl cellulose as a proton conducting biopolymer electrolyte for application in rechargeable proton battery. Electrochim. Acta.

[56] Koochakzaei A., Ahmadi H., Achachluei M. (2016). An experimental comparative study on silicone oil and polyethylene glycol as dry leather treatments. J. Am. Leather Chem. Assoc..

[57] Liu G. (2015). Advances in the theoretical understanding of photon upconversion in rare-earth activated nanophosphors. Chem. Soc. Rev..

[58] DeRosa M. (2002). Photosensitized singlet oxygen and its applications. Coord. Chem. Rev..

[59] Lee N., Cho A., Park S., Lee J.W., Sung Taek P., Park C.H., Choi Y.H., Lim S., Baek M.K., Kim D.Y. (2018). SERPINB2 is a novel indicator of stem cell toxicity. Cell Death Dis..

[60] Nicolay N., Rühle A., Perez R., Trinh T., Sisombath S., Weber K.J., Ho A.D., Debus J., Saffrich R., Huber P.E. (2016). Mesenchymal stem cells are sensitive to bleomycin treatment. Sci. Rep..

[61] Guller A., Generalova A., Petersen E., Nechaev A., Trusova I.A., Landyshev N., Nadort A., Grebenik E., Deyev S.M., Shekhter A.B. (2015). Cytotoxicity and non-specific cellular uptake of bare and surface-modified upconversion nanoparticles in human skin cells. Nano Res..

[62] Bastos V., Oskoei P., Andresen E., Saleh M.I., Rühle B., Resch-Genger U., Oliveira H. (2022). Stability, dissolution, and cytotoxicity of NaYF_4_-upconversion nanoparticles with different coatings. Sci. Rep..

[63] Tezuka K., Umezawa M., Liu T.I., Nomura K., Okubo K., Chiu H.-C., Kamimura M., Soga K. (2021). Upconversion luminescent nanostructure with ultrasmall ceramic nanoparticles coupled with Rose Bengal for NIR-induced photodynamic therapy. ACS Appl. Bio Mater..

[64] Li R., Ji Z., Dong J., Chang C.H., Wang X., Sun B., Wang M., Liao Y.-P., Zink J.I., Nel A.E. (2015). Enhancing the imaging and biosafety of upconversion nanoparticles through phosphonate coating. ACS Nano.

