# Supplementary material for: Rose Bengal-Modified Upconverting Nanoparticles: Synthesis, Characterization, and Biological Evaluation
Source: Life (Basel). 2022 Sep 5;12(9):1383. doi: 10.3390/life12091383 (PMC9502678; doi:10.3390/life12091383)
Supplement: Supplementary file 1 [file life-12-01383-s001.zip › life-1881399-supplementary.pdf]

Supplementary Materials

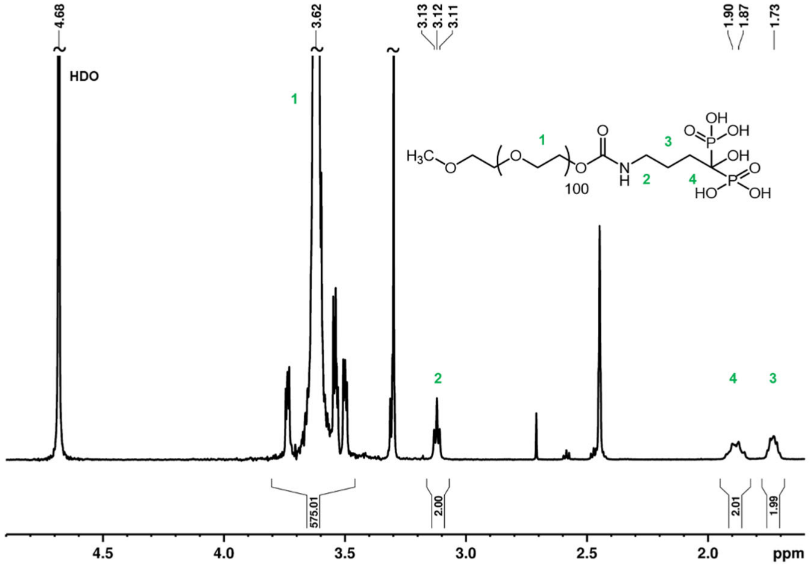

Figure S1. High-resolution ^{1}H NMR spectrum of PEG$_{5,000}$-alendronate in D_2O at 25 °C.

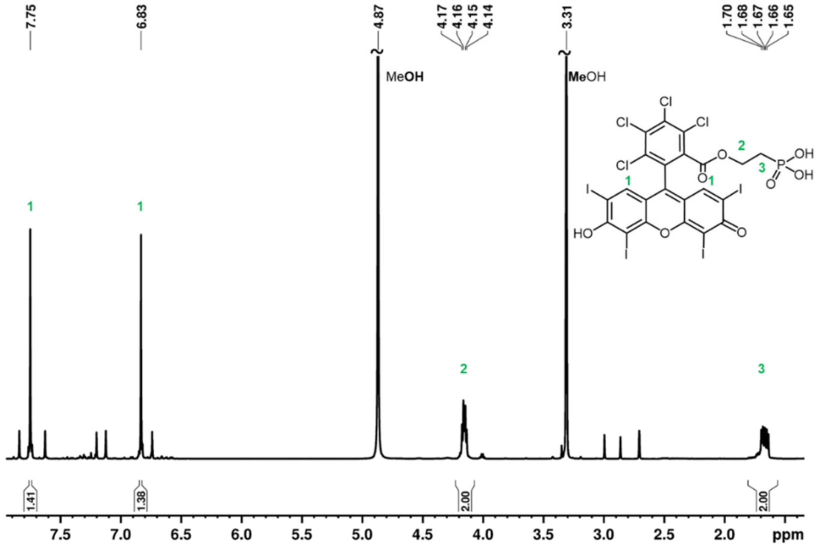

Figure S2. High-resolution ^{1}H NMR spectrum of Rose Bengal-ethylphosphonic acid in MeOD at 25 °C.

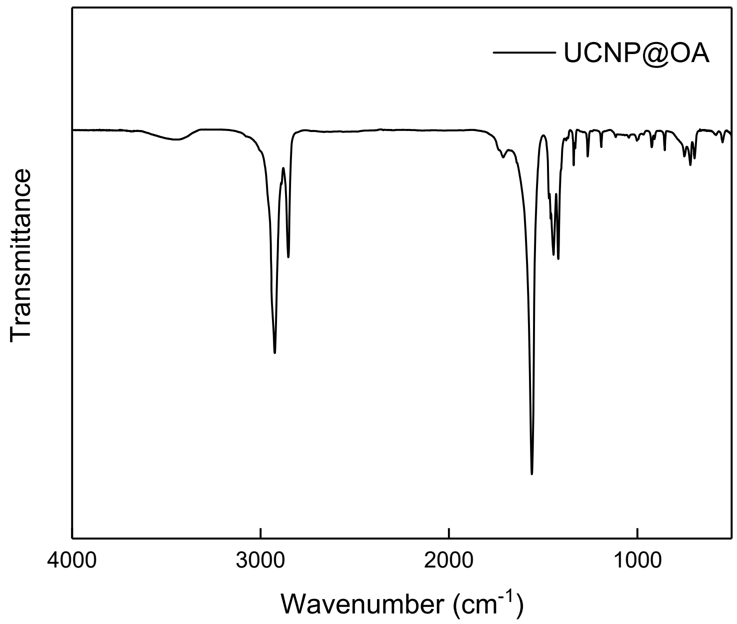

Figure S3. FTIR spectrum of UCNPs@OA.

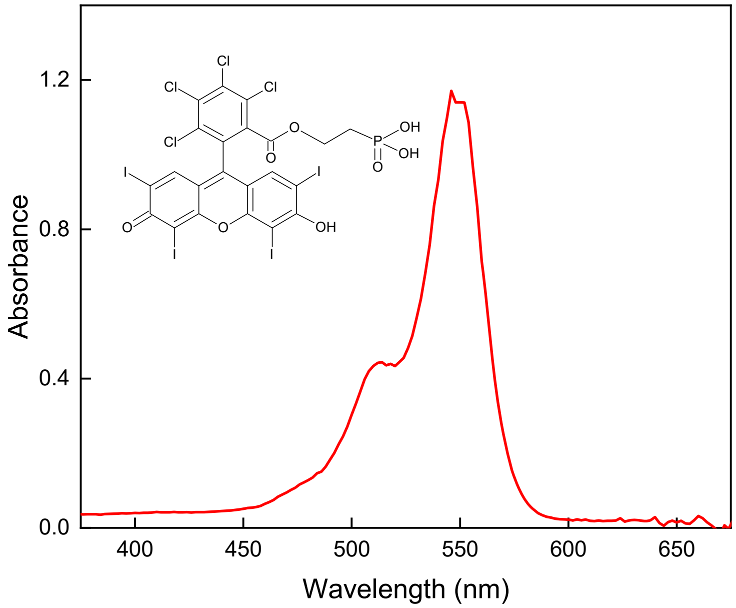

Figure S4. UV-Vis absorption spectrum of Rose Bengal-ethylphosphonic acid.

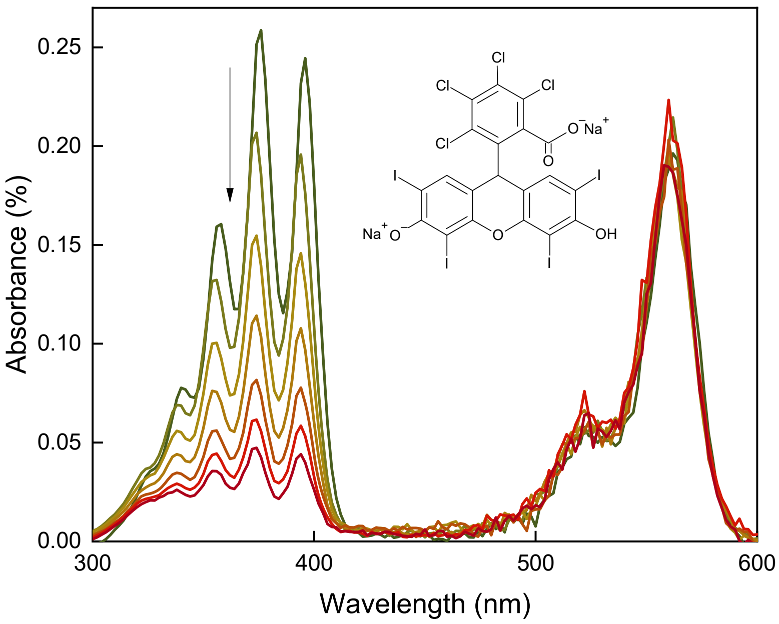

Figure S5. UV-Vis spectra of RB excited at 525-535 nm documenting time-dependent decrease of DPA absorbance due to $^1O_2$ generation. Each curve was measured after 10-min delay.
